# Biodegradation of Juglone by *Xanthomonas arboricola* pv. *juglandis*, the Causal Agent of Walnut (*Juglans regia* L.) Bacterial Blight

**DOI:** 10.1002/cbdv.202403299

**Published:** 2025-04-28

**Authors:** Marco Scortichini, Antonio Fiorentino, Elvira Ferrara, Milena Petriccione, Brigida D'Abrosca

**Affiliations:** ^1^ Research Centre for Olive, Fruit and Citrus Crops Council for Agricultural Research and Economics Roma Italy; ^2^ Department of Environmental Biological and Pharmaceutical Sciences and Technologies DiSTABiF University of Campania Luigi Vanvitelli Caserta Italy; ^3^ Research Centre for Olive, Fruit and Citrus Crops Council for Agricultural Research and Economics Caserta Italy

**Keywords:** HPLC, *Juglans regia* L. cv Sorrento, juglone, NMR, *Xanthomonas arboricola* pv. *juglandis*

## Abstract

*Xanthomonas arboricola* pv. *juglandis* is the causal agent of walnut blight, a disease affecting *Juglans regia* L. cultivations by causing severe economic losses worldwide. The content of phenolic compounds in *J. regia* cultivars plays an important role in determining the resistance or susceptibility to the infection. In this study, the assessment of antimicrobial activity against several phytopathogenic *X. arboricola* and *Pseudomonas* strains revealed that *X. a*. pv. *juglandis* was less susceptible to juglone at higher concentrations, suggesting a potential resistance mechanism. To explore the antibacterial results, the biodegradation of juglone in the presence of *X. a*. pvs. *juglandis* and *pruni* were assessed at three different collecting times by NMR. The NMR analysis clearly showed the ability of *X. a* pv. *juglandis* NCPPB1659 to degrade juglone. On the contrary, X. *a*. pv. *pruni* NCPPB2588 was ineffective. The seasonal variation of juglone collected in leaves and husks of walnuts was determined.

## Graphical abstract

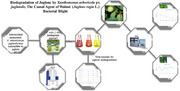

## 1 Introduction

The bacterial genus *Xanthomonas* encompasses many species and pathovars capable of inciting severe damage to cultivated crops worldwide. The phytopathogenic species *Xanthomonas arboricola* includes up to nine pathovars, two of them affect nut crops. The pathovars *juglandis*, and pv. *corylina*, indeed represent the causal agents of the bacterial blight of walnut and hazelnut, respectively [1]. The walnut blight, a disease affecting Persian (English) walnut (*Juglans regia* L.) cultivations, caused severe economic losses worldwide [2, 3]. The main symptoms include necrotic spotting on leaves and shoots, necrosis of pericarp, darkening of mesocarp and endocarp, and premature fruit drop [4]. In some cases, it causes also the so‐called “vertical oozing canker” [5]. During spring, from dormant buds *X. a*. pv. *juglandis* colonize the leaves, non‐lignified shoots, and husks and, depending on climatic conditions (i.e., air humidity, temperature) and cultivar susceptibility, incites damages to the crop [6]. Main points of plant entry are the stomata, hydathodes of trichomes, nectaria, leaf scars, and trunk lenticels [7].

The content of phenolic compounds found in leaves, shoots, and husks of *J. regia* cultivars plays an important role in determining the extent of the infection during the vegetative season. In particular, it has been shown that the seasonal alteration in the juglone (5‐hydroxy‐1,4‐naphtoquinone) content exerts a strong influence on the disease development and severity of walnut blight, indeed, in some walnut cultivars, a relevant decrease in the juglone content of the fruit at the beginning of summer is connected to a higher severity of the disease. Moreover, some *X. a*. pv. *juglandis*‐sensitive cultivars can display a higher pre‐infection juglone content in the fruit compared with the more tolerant cultivars [8]. Other studies report a higher content of juglone in the apical part of the fruit in the resistant walnut cultivar ‘Parisienne’ in comparison with the more sensitive Franquette' [9]. In young shoots, the juglone content increases during the vegetative season in the less susceptible cultivars, whereas an opposite trend was observed for the very sensitive ones [10].

These studies clearly support the hypothesis that juglone, together with other phenolic compounds, is strictly involved in the interaction with *X. a*. pv. *juglandis* during the walnut infection and represents one of the main compounds involved in the plant defense against *X. a*. pv. *juglandis*. It should be also said that *J. regia* leaves contain several phenolic compounds showing antimicrobial activity towards human bacteria [11, 12] and fungi [13, 14, 15] and that juglone per se, in addition to the well‐known allelopathic properties [16], showed a remarkable bactericidal activity to the plant pathogen *Erwinia amylovora* by showing a minimum inhibitory concentration (MIC) of 2.5–10.0 µM [17].

X. *arboricola* strains, including the pathovar *juglandis*, are supposed to cause specific plant disease mainly through different repertoires of the Type III secretion system effector proteins that overcome the defense system of the plant so that they are retained the main virulence factors of *Xanthomonas* [18, 19]. Many pathogenic strains of this pathovar also possess mobile genetic elements (i.e., transposons and genomic islands) which contain genes related to copper and antibiotic resistance as well as genes capable of conferring adaptation to the plant environment [20]. In addition, the de novo synthesis of arginine is required for displaying the full virulence toward the host plant [21].

Recently a new strain of *Xanthomonas* sp., namely CPBF424, was isolated from the asymptomatic dormant buds of a diseased walnut tree in Portugal [1, 22, 23]. According to the multilocus sequence analysis, this new strain is located between the nonpathogenic *X. arboricola* and *X. prunicola* clusters. Though *Xanthomonas* sp. CPBF424 is divergent from *X. a*. pv. *juglandis*, pathogenicity tests confirmed that CPBF424 is pathogenic to walnut trees. Based on these data Xanthomonas pathoadaptations may be held responsible for walnut pathogenicity [23].

However, in the case of *X. a*. pv. *juglandis* the occurrence of phenolic compounds in *J. regia* tissues would seem to represent a relevant barrier to face for starting the infection in leaves, shoots, and husks.

The present study aims to evaluate the antimicrobial activities of juglone against several phytopathogenic bacteria. Starting with *X*. *a*. pv. *juglandis*, the least susceptible bacterium compared to other phytopathogenic species, we have assessed whether *X*. *a*. pv. *juglandis* can biodegrade juglone in vitro.

Simultaneously, was evaluated the juglone content (both in leaves and green husks) in walnut cultivar ‘Sorrento’, the landrace mainly presents in the south of Italy [24], during several phenological stages. This is the first study that shows how the unique capability to degrade juglone confers the possibility to *X. .a* pv. *juglandis* of colonizing Persian walnut leaves and green husk, thus starting the infection process.

## 2 Results and Discussion

### 2.1 Antibacterial Activity Evaluation

The MIC of juglone against various *X. arboricola* and *Pseudomonas* strains was determined (Table 1). At low concentrations until 0.1 mM, juglone shows no growth inhibition for all the tested bacterial strains, meaning the bacteria grow normally at these concentrations. At 10 mM, strains such as *X. a*. pv. *pruni*, *X. a*. pv. *fragariae*, *Pseudomonas avellanae*, *Pseudomonas syringae* pv. *actinidiae*, *Pseudomonas viridiflava*, and *P. s*. pv. *syringae* exhibit growth inhibition. Notably, *X. a*. pv. *juglandis* continues to growth until 10 mM, suggesting that it is less susceptible to juglone at higher concentrations compared to the other strains (Table 1).

**TABLE 1:** Bacterial growth responses to different juglone concentrations after 24 h as observed in some *Xanthomonas arboricola* pathovars and some phytopathogenic *Pseudomonas* species strains.

	Concentration of juglone (mM)							
Bacteria	0.001	0.01	0.1	1	10	12.5	25	50
*Xanthomonas arboricola* pv. *juglandis* NCPPB1659	+	+	+	+	+	−	−	−
*Xanthomonas arboricola* pv. *pruni* NCPPB2588	+	+	+	+	−	−	−	−
*Xanthomonas arboricola* pv. *fragariae* PD2780	+	+	+	+	−	−	−	−
*Pseudomonas avellanae* CRA‐PAV013	+	+	+	+	−	−	−	−
*Pseudomonas syringae* pv. *actinidiae* CRA‐FRU 8.43	+	+	+	+	−	−	−	−
*Pseudomonas viridiflava* NCPPB1382	+	+	+	+	−	−	−	−
*Pseudomonas syringae* pv. *syringae* CRA‐FRU10.31	+	+	+	+	−	−	−	−

*Note*: ‘+’ indicates observed growth of bacteria, ‘−‘ indicates no visible growth of bacteria.

Several studies have demonstrated phenolic compounds can lower extracellular pH, contributing to their antibacterial activity. These compounds disrupt bacterial membranes, leading to the leakage of nucleic acids, proteins, and ions such as potassium and phosphate, thereby compromising both membrane integrity and cytoplasmic function [25].

The antibacterial efficacy of juglone has been extensively evaluated in vitro [26, 27]. Studies indicate that juglone exhibits minimal degradation at low microbial concentrations after 24 h of incubation [27, 28]. Moreover, the compound remains stable in solutions with pH values ranging from 2.0 to 8.0 over a 24‐h period [29]. Juglone exhibited strong antibacterial activity against *Escherichia coli* and *Staphylococcus aureus*, with a MIC value of 15.625 µmol/L [11]. Juglone induces oxidative damage to *E. coli* DNA by promoting the production of reactive oxygen species (ROS). It also inhibits RecA protein expression, suppressing the SOS response and reducing DNA repair ability in *E. coli* [11]. It exhibited an even lower MIC of 50 µg/mL against *Listeria monocytogenes* [30]. Despite limited research on phytopathogenic bacteria, juglone shows strong bactericidal activity against *E. amylovora* with a MIC of only 2.5–5 µM at pH 4.5 [17] and *Psedomonas gingeri*, *P*. *syringae* pv. *phaseolicola* (wild types) [31]. Mechanistic studies revealed that juglone induces bacterial cell death by generating ROS, which subsequently causes DNA damage [11]. Wang et al. [32] used a proteomic approach, to demonstrate that juglone inhibits key pathways involved in protein synthesis, the tricarboxylic acid cycle, and nucleic acid synthesis in *S. aureus*.

We further focused on investigating the reduced susceptibility of juglone on phytopathogenic bacterium *X. arboricola* pv. *juglandis*.

Likewise, the three most economically important pathovars within *X. arboricola* are pathovars *pruni*, *corylina*, and *juglandis*, highly phylogenetically related [1] and responsible for bacterial spot of stone fruit trees, bacterial blight of hazelnut, and walnut blight, respectively [33]. Based on literature evidence that some bacteria can degrade juglone in soil and *Pseudomonas* J1 can use juglone as their sole carbon source [34], in this study the degradation of juglone in the presence of *X*. *arboricola* *pvs*. *juglandis* (NCPPB1659 and CREA‐DC1018) and *pruni* NCPPB2588 (Table 2) was evaluated by NMR respect to control, namely juglone‐containing media without bacteria.

**TABLE 2:** Selected *Xanthomonas arboricola* pathovars strains used in this study to evaluate the capability to degrade juglone.

Pathogens	Code	Host	Country of origin
*X. arboricola* pv. *Juglandis* (XaJ)	NCPPB1659	*Juglans regia*	United Kingdom
*X. arboricola* pv. *Juglandis* (XaJ)	CREA‐DC1018	*Juglans regia*	Italy (Latium)
*X. arboricola* pv. *pruni* (XaP)	NCPPB2588	*Prunus persica*	South Africa

The ability of *X. arboricola* pvs. *juglandis* and *pruni* to biodegrade juglone were evaluated at three different times (24, 48 and 72 h). After incubation, the culture media were collected, extracted with Et_2_O and the organic phase, subsequently analyzed by NMR.

**FIGURE 1:**
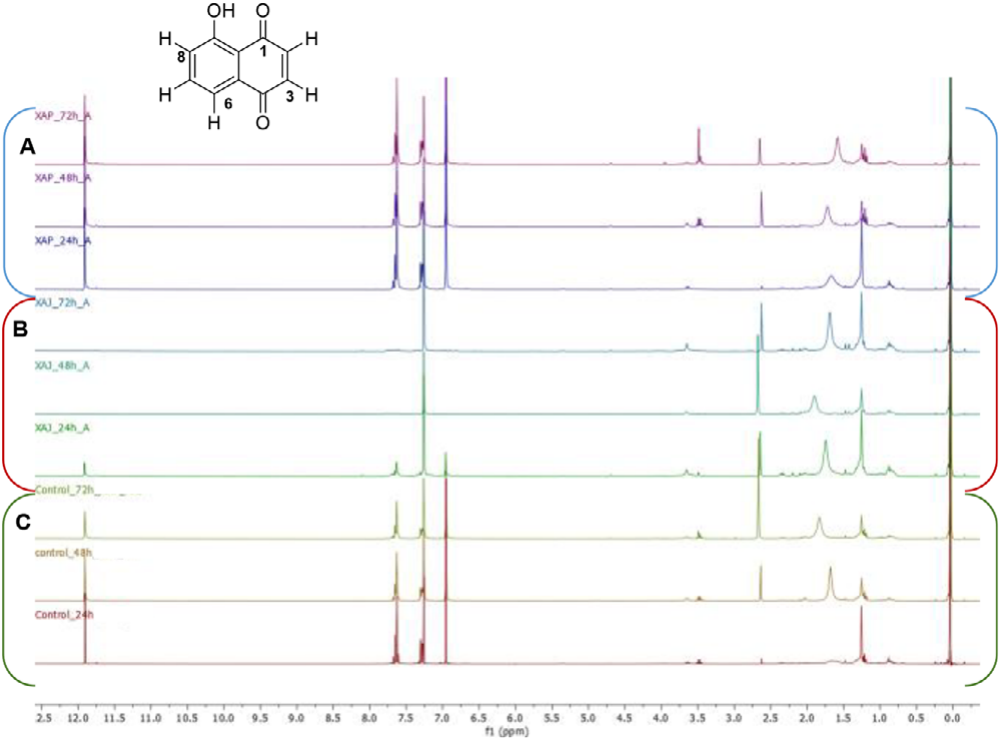
^1^H NMR spectrum of the culture media recorded in CDCl_3_ with and without bacterial strains (control, Panel C). XAP, *Xanthomonas arboricola* pv. *pruni* NCPPB2588 (Panel A). XAJ, *Xanthomonas arboricola* pv. *juglandis* NCPPB1659 (Panel B).

^1^H NMR spectrum of control as well as of *X. a*. pv. *pruni* NCPPB2588 (Figure 1) showed typical signals of 5‐hydroxy‐1,4‐naphtoquinone, as a singlet at *δ*_H_ 6.95 related to two protons of a quinoid moiety, the double doublet at *δ*_H_ 7.29 (H‐6) as well as a multiplet at *δ*_H_ 7.64 (H‐7/H‐8) associated to the aromatic ring.

Otherwise, inspection of ^1^H NMR of organic phase obtained from *X. a*. pv. *juglandis* NCPPB1659, culture inoculated with juglone, clearly showed the ability of *X. a*. pv. *juglandis* NCPP1659 to degrade juglone, with a marked degradation of naphtoquinone derivative after 48 h. Correspondingly, the color of these latter fractions faded away along with the degradation process, indicating the chromophore in juglone was destroyed. However, the inspection of ^1^H NMR spectra not revealed other signals that could suggest any products obtained from biodegradation of juglone. Furthermore, the absence of signals in aromatic region, not hypnotized the biotransformation of juglone in other derivatives as instead reported in literature data associated to biodegradation of lawsone by *Pseudomonas putida* L2 [35]. Salicylic acid, 2‐hydroxy‐4‐oxo‐chroman‐2‐carboxylic acid, and catechol, in fact, have been elucidated as the potential intermediates of lawsone biodegradation pathway, a structural isomer of juglone.

**FIGURE 2:**
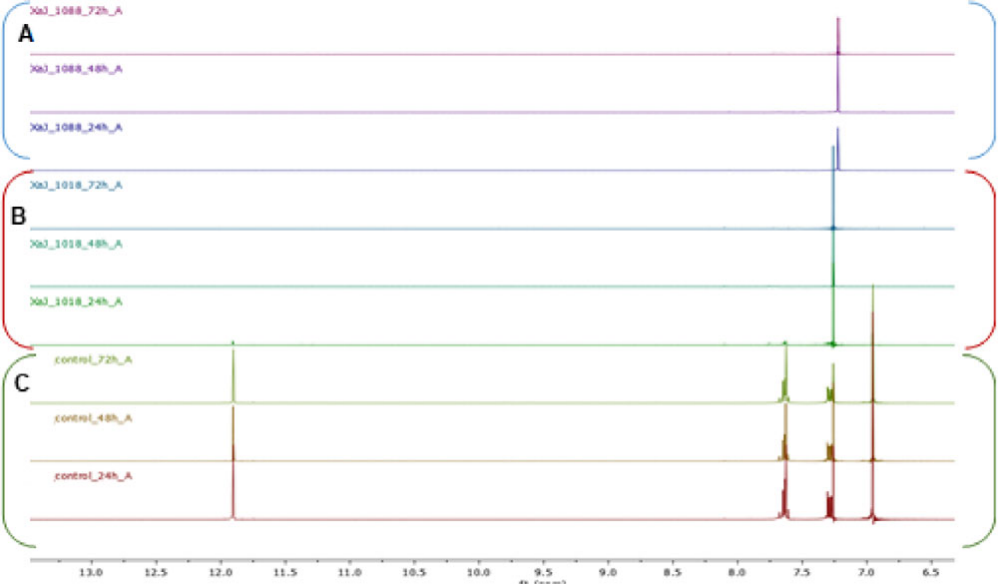
Expanded region of ^1^H NMR spectrum of the culture media recorded in CDCl_3_ with and without bacterial strains (control, Panel C). *Xanthomonas arboricola* pv. *juglandis* NCPPB1659 (Panel A). *X. arboricola* pv. *Juglandis* CREA‐DC1018 (Panel B).

To better investigate the capability of *X. a*. pv. *juglandis* to biodegrade juglone, two strains, namely NCPPB1659 and CREA‐DC1018, have been incubated in the presence of juglone. The bleaching of the medium, as well as ^1^H NMR analyses, suggested a time‐dependent biodegradation of juglone, operated by both strains and a better efficiency to biodegrade this compound exerted by NCPPB1659 (Figure 2).

**FIGURE 3:**
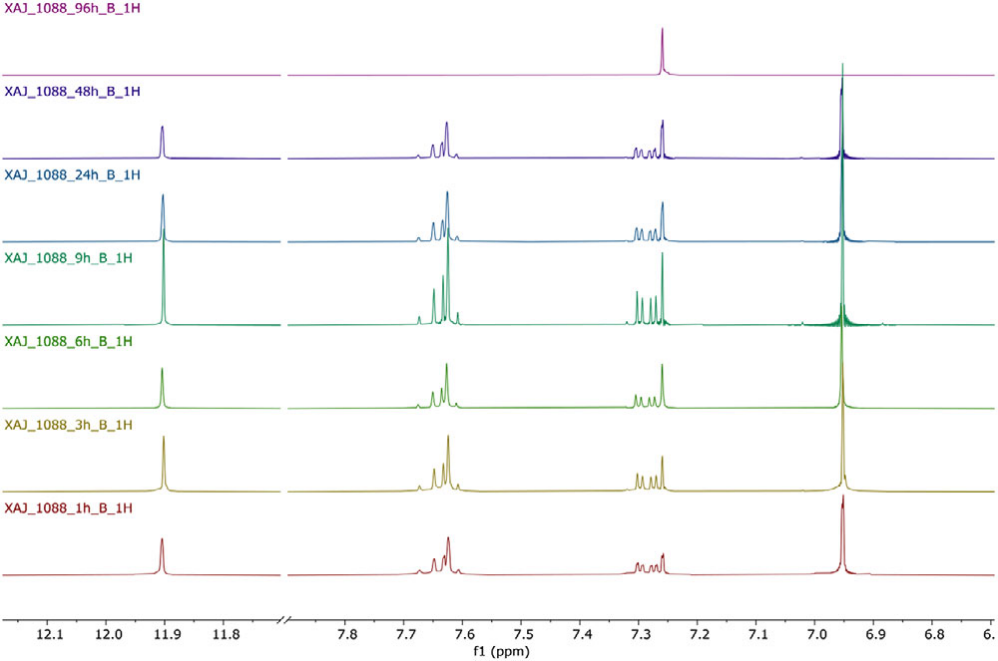
Expanded region of ^1^H NMR spectrum of the culture media with *Xanthomonas arboricola* pv. *juglandis* NCPPB 1659.

**FIGURE 4:**
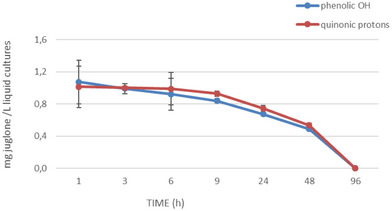
Juglone biodegradation in the presence of: *Xanthomonas arboricola* pv. *juglandis* NCPPB1659 detected by NMR.

To further investigate the half‐life of juglone, additional experiments have been performed. The ability of *X. a*. pv. *juglandis* NCPPB1659 to degrade juglone was evaluated at seven different times (1, 3, 6, 9, 24, 48 and 72 h). The progressive bleaching of samples and NMR analysis (Figure 3) of same drawn at various times showed a time‐dependent disappearance of juglone. The integration of buckets corresponding to quinonic protons as well as to hydroxyl group suggested 48 h as the half‐life of juglone (Figure 4).

Literatura data confirm that juglone undergoes degradation also by the ligninolytic fungus *Pleurotus sajor‐caju* [36].

### 2.2 HPLC Analysis of Juglone in Walnut Leaves and Green Husks

**FIGURE 5:**
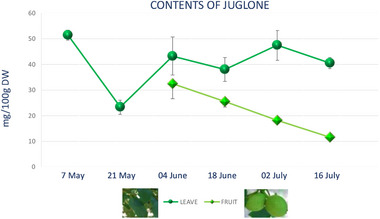
Juglone content in walnut green husks and leaves. Data are expressed as mean values ± SD (mg/100 g dry weight).

The seasonal variation of juglone in walnut leaves and green husks, collected for 3 months, at six sampling dates was determined. The results presented in Figure 5 showed the highest juglone content in walnut leaves on May 7 (51.453 mg/100 g dry weight) following by July 3 sampling date, when the juglone content was 47.4/100 g FW. The lowest content has been revealed at the end of May 23 (3/100 g FW).

Our data are in good agreement with those reported by Thakur [37] related to analyses of 1121 tree leaf samples that highlighted juglone variation in the range of 13.1–1556.0 mg/100 g dry weight.

During the growing season, there were oscillations of mean juglone content [38]. In fact, previous research on juglone content in walnut leaves, by the end of the vegetation period, showed that its content ranged between 5.42 and 22.82 mg/100 g in different analyzed cultivars [39]. So, our experimental results indicated that walnut leaves contain a concentration of juglone, comparable to those obtained in previous studies [40].

Likewise, in black walnut leaves, a linear decrease of juglone over the growing season has been detected [41].

In green husks, the peak of juglone content was determined at the beginning of June (32.487 mg/100 g dry weight), and from here, it steadily decreases until July 16 (11.613 mg/100 g dry weight). The graph shows the variation of the juglone between June and July since the fruit in May is not yet present (Figure 5). According to literature data, juglone, a well‐known component of walnuts, is found in considerable amounts in all green and growing parts of trees and unripe hulls of nut. Stampar et al. [42] found a juglone content of 1404 mg/100 g dry weight in fresh walnut husks on June 21 and only 218 mg/100 g dry weight on August 19.

Measurements of seasonal distribution of juglone among various tissues of pecan revealed that the highest concentrations occurred in leaflets in June and in nuts in September [43].

The concentrations of phenols like juglone depend on the developmental stage of nuts [10] and change in ecological parameters [40]. As regards correlation between content and time period, the highest content of phenolic compounds was found in May and July. Agreeing to the data juglone is more concentrated in the leaves than in green husks.

Literature data highlighted a great variability in the content of different phenolic compounds across different cultivars (Table 3) and developmental stage. In addition, it emphasized that different cultivars exhibit varying responses to the same infection, suggesting that a broader range of cultivars is necessary when studying a plant's response.

**TABLE 3:** Selected literature data about juglone content across different *Juglans regia* cultivars **.**

Bacterial strains	Investigated cultivars	Organ of plants	Phenolic compounds detected	Content of juglone	Evaluation of seasonal changes	Ref
*X. arboricola* pv. *juglandis*	Cisco, Elit, Fernor, Franquette, Hartley, Šampion	GH	13	1.128–3.363 mg/100 g1 of dry weight	Yes	[44]
*X. arboricola* pv. *juglandis*	Cisco, Elit, Fernor, Franquette Hartley, Šampion	S	8	Juglone and 1,4‐naphthoquinones 71–528 mg/100 g of dry weight	Yes	[10]
*X. arboricola* pv. *juglandis*	Fernor, Fernette, Franquette Krka Rubina Sava	HL	52	170–800 mg/100 g of dry weight	No	[45]
*X. arboricola* pv. *juglandis*	Fernor, Fernette, Franquette Krka Rubina Sava	IL	52	73–901 mg/100 g of dry weight	No	[45]
*X. arboricola* pv. *juglandis*	Cisco, Franquette, Šampion	GH	7	438–5075 mg/100 g of dry weight	Yes	[8]

Abbreviations: GH, green husks; HL, healthy leaves; IL, infected leaves; S, annual shoots.

## 3 Conclusions

Although the antibacterial activities of juglone have been extensively evaluated against human pathogens, unlike a few papers investigate phytopathogenic bacteria.

The results reported in this paper increase knowledge of the antibacterial activity of juglone against phytopathogenic bacteria selection, *X. a*. pv. *juglandis* as less susceptible to juglone at higher concentrations. In order to explain these results, the ability of *X. a*. pv. *juglandis* to degrade juglone has been investigated. The NMR analysis highlighted the ability of *X. a*. pv. *juglandis* NCPPB1659 to degrade juglone. The progressive bleaching of samples and NMR analysis of the same drawn at various times showed a time‐dependent disappearance of juglone.

This study provides insights into the role of juglone in walnut tree defense and its bactericidal effects and also shows how the capability of degrading it confers to *X. a*. pv*. juglandis* the possibility to initiate the infection process to Persian walnut leaves and green husks.

The effective management of walnut blight is rather difficult since this phytopathogen can face the antibacterial activity of copper compounds through the wide occurrence in its populations of copper‐resistant genes. Its capability to degrade juglone adds other difficulties for the walnut blight control of Persian walnut orchards. A weather‐based model based on the bacterium epidemiology can help to predict the outcome of the infection for a more accurate timing of bactericides distribution [46].

Related bacterial species such as *Xanthomonas oryzae* produces a PSY1‐like sulfated peptide to reducing stress responses and favoring growth and development [47].

The searching of resistance or tolerant traits to *X. a*. pv. *juglandis* in the wild *J. regia* germplasm populations could offer the possibility to introduce such a feature in new cultivars to possibly reduce or eliminate the distribution of bactericides in the environment [45].

## 4 Experimental Section

### 4.1 Chemicals and Reagents

All the solvents and hexamethyldisilane were purchased from Sigma‐Aldrich (Milano, Italy). Agar was furnished by Oxoid, UK.

### 4.2 Walnut Leaf and Fruit Samplings

Leaves and green husks samples of *J. regia* cv. Sorrento were collected every 14 days from May 7 to July 16. All samples were taken from 20‐year‐old walnut trees cultivated under standard commercial practices in an experimental walnut orchard in Caserta, Southern Italy (41°04′ N, 14°19′ E). This orchard, managed by the CREA—Research Centre for Olive, Fruit Trees, and Citrus, serves as a repository for walnut genetic resources. Young leaves and green husks were frozen in liquid nitrogen and kept at −80°C until further analysis.

### 4.3 Antimicrobial Activity of Juglone

To evaluate the antimicrobial activity of juglone, the following bacterial strains *X. a*. pv*. juglandis*, *X. a*. pv*. pruni*, *X. a*. pv. *fragariae*, *P. avellanae*, *P. s*. pv*. actinidiae*, *P. viridiflava* and *P. s*. pv. *syringae* were used (Table 1). All bacterial strains were cultured in nutrient broth at 24°C for 18–24 h until an optical density at 600 nm (OD_600_) of 0.1 was reached. Bacterial cultures were then inoculated with juglone at different concentrations, ranging from 10^−6^ M to 10^−2^ M in 1:10 serial dilutions. Juglone was dissolved in dimethyl sulfoxide (DMSO) before being added to the cultures. The inoculated cultures were incubated in a rotary shaker at 24°C and 200 rpm for 72 h. Solvent control assays (containing DMSO without juglone) were always conducted in parallel. Microbial growth was assessed after 24 and 72 h by plating on nutrient agar (Oxoid, Milan, Italy) supplemented with 3% (w/v) sucrose (NSA). The MIC of juglone was determined based on bacterial growth after 24 h of incubation. Bacterial suspensions without juglone served as positive controls, while nutrient broth containing juglone (without bacteria) was used as a negative control.

### 4.4 Statistical Analysis

All experiments were performed in triplicate, and results were analyzed using one‐way analysis of variance (ANOVA), followed by Tukey's post hoc test to determine significant differences between treatments (*p* < 0.05). Data were expressed as mean ± standard deviation (SD).

### 4.5 *X. arboricola* Strains and Methodology

Two strains of *X. a*. pv. *juglandis* (NCPPB1659 and CREA‐DC1018) and one strain of *X. a*. pv. *pruni* (NCPPB 2588), originally isolated from *Prunus persica*, were used to assess juglone biodegradation. The bacterial strains were cultured on nutrient agar (Oxoid, UK) supplemented with 3% (w/v) sucrose (NSA) and incubated at 25 ± 1°C for 48 h.

For liquid culture experiments, a bacterial suspension was prepared by adjusting the cell density to 1 × 10^4^ colony‐forming units per milliliter (cfu/mL) in sterile nutrient broth (13 g/L, Oxoid, UK). The suspensions were transferred into 1000‐mL Erlenmeyer flasks and placed on an orbital shaker incubator at 24°C and 100 rpm for 24 h until an optical density at 600 nm (OD_600_) of 0.1 was reached.

Following bacterial cultures were inoculated with a 1 mM juglone solution, which had been previously dissolved in dimethyl sulfoxide (DMSO). The inoculated cultures were then incubated at 24°C and 200 rpm in a rotary shaker for 72 h. Parallel solvent control assays, containing DMSO without juglone, were performed under the same conditions.

To monitor juglone biodegradation, bacterial cultures were collected at 24, 48 and 72 h after juglone addition. In addition, a more detailed degradation study was conducted using *X. a*. pv. *juglandis* NCPPB1659, with samples collected at 1‐, 3‐, 6‐, 9‐, 24‐, 48‐ and 72‐h post‐inoculation.

All experiments were performed in triplicate to ensure reproducibility. The collected samples were subsequently processed for further analysis to quantify juglone degradation.

### 4.6 Extraction and Identification of 5‐Hydroxy‐1,4‐Naphtoquinone

**SCHEME 1:**
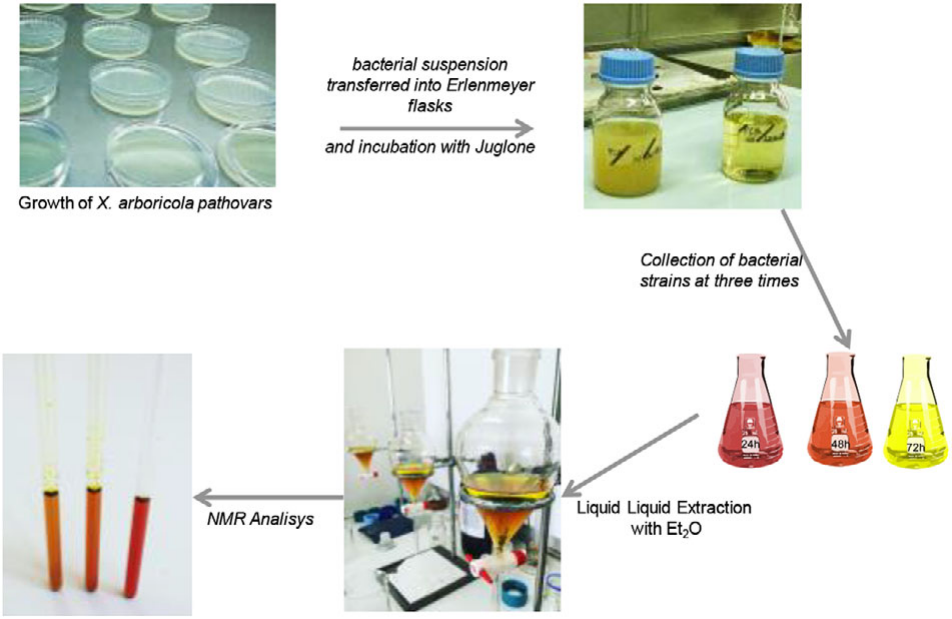
Experimental workflow.

Fifty milliliters of culture media (three replicates for each treatment) containing strains and juglone or medium with alone juglone was extracted by liquid–liquid extraction. In particular, 50 mL of Et_2_O was added to culture media and then shaken by liquid–liquid extraction using Et_2_O as extracting solvent (two times), obtaining an organic (Fraction A) and an aqueous fraction (Fraction B). Both organic fractions gathered, dried with Na_2_SO_4_, were concentrated under vacuum and stored at −20°C until NMR analysis (Scheme 1).

The dried sample was dissolved in 650 µL of CDCl_3_ containing 0.1% w/w hexamethyldisilane and transferred to a standard 5‐mm NMR tube for NMR analysis. NMR spectra were recorded at 25°C on a 300.03 MHz for ^1^H and 75.45 MHz for ^13^C on a Bruker AVANCE II 300 MHz NMR Spectrometer Fourier transform. CDCl_3_ was used as the internal lock. Each ^1^H NMR spectrum consisted of 256 scans with the following parameters: 0.16 Hz/point, acquisition time (AQ) = 1.0 s, relaxation delay (RD) = 1.5 s, 90° pulse width (PW) ¼ 13.8 ms. FIDs were Fourier transformed with LB = 0.3 Hz.

### 4.7 Time Courses for the Biodegradation and Quantification of 5‐Hydroxy‐1,4‐Naphtoquinone

To further investigate time‐dependent biodegradation for the juglone additional experiments have been performed. Culture media containing juglone with or without *X. a*. pv. *juglandis* NCPPB1659 were collected at seven different times (1, 3, 6, 9, 24, 48 and 72 h, three replicates per time point). Fifty milliliters of each sample (was extracted by liquid–liquid extraction using Et_2_O as extracting solvent as previously described. The organic fractions, dried with Na_2_SO_4_, concentrated under vacuum were dissolved in 650 µL of CDCl_3_ containing 0.1% w/w hexamethyldisilane and transferred to a standard 5‐mm NMR tube for NMR analysis. The NMR spectra were recorded as described above in the previous paragraph. The resulting spectra of three replicates per timepoint were manually phased and baseline‐corrected and calibrated to hexamethyldisilane at 0.0 ppm using ^1^H NMR processor ACDLABS 12.0. ^1^H NMR spectra were bucketed, reducing it to integral segments with a width of 0.02 ppm with ACDLABS 12.0^1^H NMR processor (ACDLABS 12.0, Toronto, Canada). The buckets corresponding to non‐overlapping signals were manually integrated, scaled to the internal standard signal, and used to calculate the relative amount of juglone. Results were expressed as mg (±SD) of metabolite for 1 L of bacterial medium.

### 4.8 HPLC Analysis of Juglone in Walnut Leaves and Green Husks

Fresh leaves were collected in six sampling dates in the year (May 7, May 21, June 4, June 18, July 2, and July 16).

The samples were prepared according to Solar et al. [10]. Dried leaves and green husks material was powdered. Fifteen milligrams of finely ground powder was extracted by ultrasound‐assisted extraction (Elma Transonic Digitals), with 1.5 mL of methanol for 45 min at room temperature. After extraction, the treated samples were centrifuged for 5 min at 4200 rpm. The supernatant was separated from the pellet and solubilized in a total of 2 mL of methanol, then filtered on a millex, transferred into a vial and analyzed with HPLC on the same day, further, to avoid the problems of preservation [24]. HPLC analysis was performed using the Shimadzu HPLC system (Shimadzu Corporation, Kyoto, Japan) which included a system controller (CBM‐20A), a pumping system (LC‐20AD), a photodiode array detector (SPD‐M20A) and a 20 µL loop. The HPLC analysis was performed on a Supelco Ascentis C‐18 column (250 × 4.6 mm, 5 µm). The solvent system is a solvent gradient A (water and 2% acetic acid) and solvent B (acetonitrile): initial 10% B, linear 10%–55% in 45 min, 55%–100% in 60 min, and from isocratic to 10% in 5 min to rebalance the column. The injection volume was 20 µL at a flow rate of 1.0 mL/min. The first chromatogram obtained is related to the standard (juglone) and shows a peak (*t*_R_ = 50.6 min). These preparations are carried out to determine retention times and then identify the compound in the extract. By injecting the juglone at various concentrations, it was possible to obtain a calibration line that allows us to quantitatively analyze the concentration of the juglone inside the extracts of leaves and green husks.

## Author Contributions

**Marco Scortichini**: methodology, writing – original draft preparation, writing – review and editing, supervision. **Antonio Fiorentino**: conceptualization, methodology, writing – review and editing, supervision. **Elvira Ferrara**: investigation. **Milena Petriccione**: conceptualization, writing – review and editing. **Brigida D'Abrosca**: conceptualization, methodology, investigation, writing – original draft preparation, writing – review and editing. All authors have read and agreed to the published version of the manuscript.

## Conflicts of Interest

The authors declare no conflicts of interest.

## Data Availability

The authors have nothing to report.
